# Challenges in applying W.A.A.V.P. criteria to diagnosing triclabendazole resistance in *Fasciola hepatica*, an example from the Southern Tablelands of New South Wales, Australia

**DOI:** 10.1016/j.ijpddr.2025.100618

**Published:** 2025-09-30

**Authors:** Chelsie Uthayakumar, Hayley Martinez DeCristi, Emily Kate Francis, Roger Alan Willoughby, Shannon Taylor, Nichola Eliza Davies Calvani

**Affiliations:** a The Sydney School of Veterinary Science, Faculty of Science, The University of Sydney, New South Wales, 2006, Australia; b Gunning Ag & Water Solutions, Gunning, NSW, 2581, Australia; c The University of Sydney, NSW, 2021, Australia; d The University of Sydney Infectious Diseases Institute (Sydney ID), The University of Sydney, NSW, 2006, Australia

**Keywords:** Albendazole, Anthelmintic resistance, Closantel, Goats, Liver fluke, Ruminants

## Abstract

*Fasciola hepatica* (liver fluke) is a zoonotic parasite of global concern. In Australia, it is the 13th most important cause of economic loss in the sheep meat industry alone. Resistance to the frontline drug, triclabendazole (TCBZ), was first recorded in Australia in 1995 and has since emerged globally. In 2023, producers from the New South Wales (NSW) Southern Tablelands raised concerns over a reported 230% increase in liver fluke, which they suspected was due to drug resistance. To confirm or deny these suspicions, we co-designed a diagnostic field investigation aligned with guidelines from the World Association for the Advancement of Veterinary Parasitology (W.A.A.V.P.) to evaluate the prevalence and susceptibility of *F. hepatica* on naturally infected sheep, cattle, and goat properties. Nine mobs (seven sheep, one goat, one cattle) across eight farms were divided into three treatment groups (15 animals/group) and treated with either TCBZ, closantel/abamectin (CLOS/AVM, positive control – sheep), albendazole (ABZ, positive control – goats), or water (H_2_O; negative control). Prevalence was determined by sedimentation and faecal egg count (FEC), alongside a commercial coproantigen ELISA (cELISA) and in-house qPCR. Drug efficacy was assessed using faecal egg count reduction tests (FECRT) and coproantigen reduction tests (CRT). Four of the eight farms had a within-herd true prevalence >25%. TCBZ resistance was confirmed on one sheep property (86–89% efficacy). The goat property demonstrated susceptibility to TCBZ (97–98% efficacy), but reduced efficacy of ABZ (79%), representing the first potential report of ABZ resistance in *F. hepatica* infecting goats. Nemabiome sequencing of co-infecting gastrointestinal nematodes confirmed widespread benzimidazole resistance, underscoring the broader challenges faced by producers. Other potential causes of drug failure included climate variability, pseudo-parasites, and low cELISA diagnostic sensitivity. These results highlight the complexity of diagnosing and managing drug resistance in naturally infected populations and reinforce the need for *Fasciola*-specific W.A.A.V.P. guidelines.

## Highlights

• Farmer-led trial confirms triclabendazole resistance in *F. hepatica*.
• First potential report of albendazole resistance in *F. hepatica* in goats.
• “Drug failure” linked to climate, pseudo-parasites, and diagnostic limitations.
• Multi-modal diagnostics improved *F. hepatica* resistance interpretation.
• We call for *Fasciola-*specific W.A.A.V.P. anthelmintic resistance guidelines.

## Graphical abstract

**Figure:**
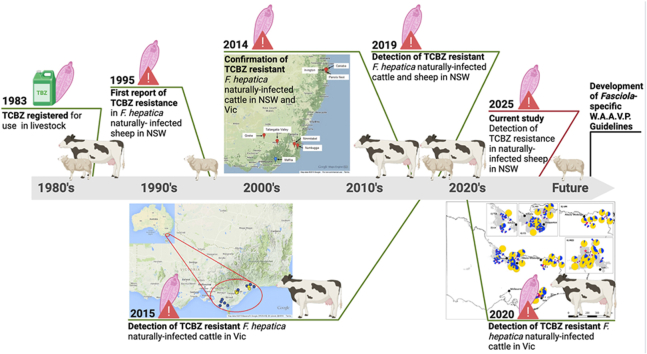

## 1 Introduction

The continued emergence and spread of *Fasciola hepatica* resistant to the frontline drug, triclabendazole (TCBZ), poses a significant challenge to the global livestock industry and human health (Kelley et al., 2016). TCBZ was first registered for use in livestock infected with *F. hepatica* in the 1980s and remains the only drug approved for treating human fasciolosis due to its unique efficacy (>99%) against both immature and adult stages (Wessely et al., 1988; Anonymous, 1998; Fairweather, 2005; Novartis, 2019). This broad range of activity against mammalian life stages has entrenched TCBZ as the drug of choice for control of *F. hepatica* in livestock, leading to its widespread use (Fairweather and Boray, 1999; Fairweather et al., 2020; Lamb et al., 2022).

The overreliance on TCBZ by livestock producers led to the first report of drug resistant *F. hepatica* in sheep in Victoria, Australia during a field study in 1995 (Overend and Bowen, 1995). Since then, cases of TCBZ resistant *F. hepatica* have been reported across multiple continents, raising serious concerns over the sustainability of chemical control in the absence of new anthelmintics under development (Gaasenbeek et al., 2001; Winkelhagen et al., 2012; Hanna et al., 2015; Larroza et al., 2023). In Australia, despite growing awareness of resistance amongst producers, a recent survey in north eastern New South Wales (NSW) reported that 38% of farmers administer liver fluke drenches without first confirming infection, reflecting a broader failure to implement strategic parasite control that likely furthers the development of resistance (Lamb et al., 2022).

The economic burden of fasciolosis in Australia is considerable. Production losses arise primarily from the damage caused by migrating immature flukes, which compromise liver function and lead to reduced growth rates, fertility, and wool quality, and, in severe cases, death (Sinclair, 1962). In sheep alone, estimated losses increased from $25 million in 2015 to $38.2 million in 2022, making liver fluke the 13th most significant endemic disease affecting meat production in Australia (Shephard et al., 2022). In the 2023/24 financial year, liver fluke was the third most common cause of condemnation in sheep in NSW (Animal Health Australia, 2025). However, these figures do not account for the substantial losses likely sustained by the beef and dairy sectors, where liver fluke infection can reduce feed conversion efficiency, delay finishing times, and affect milk yield and protein content (Schweizer et al., 2005; Charlier et al., 2014; Mazeri et al., 2017; da Costa et al., 2019). Despite these well-recognised impacts, the true economic cost of fasciolosis across all livestock sectors in Australia remains unknown. A more comprehensive assessment is urgently needed to inform the prioritisation of future research into the parasite's epidemiology, economic impact, and control strategies.

The parasite's complex life cycle involves both definitive and intermediate hosts, with a prolonged prepatent period of 8–12 weeks before the appearance of eggs in faeces, which hinders early diagnosis (Calvani et al., 2018; Fairweather et al., 2020; Lalor et al., 2021). To overcome diagnostic delays, a coproantigen Enzyme Linked Immunosorbent Assay (cELISA) was commercialised that allows detection of *F. hepatica* infection 6–8 weeks post infection (Almazan et al., 2001; Mezo et al., 2004; Calvani et al., 2018). Although issues around sensitivity have been reported in diagnosing low-burden infections, when used in parallel with traditional diagnostic methods (sedimentation and faecal egg count; FEC), cELISAs improve diagnostic accuracy and facilitate earlier detection of drug failure (Brockwell et al., 2013, 2014; Calvani et al., 2017; George et al., 2019). A combined diagnostic approach is increasingly recommended for the detection of anthelmintic resistance in field trials (George et al., 2019; Fairweather et al., 2020; Burden et al., 2024).

With few drugs registered with high efficacy against immature *F. hepatica*, and no vaccine currently available, regular anthelmintic resistance monitoring and reporting remains a critical component of sustainable liver fluke control. The current investigation was undertaken after suspicions were raised by farmers over potential drug resistance due to anecdotal reports of a 230% increase of liver fluke in livestock in the NSW Southern Tablelands between 2019 and 2022 (*Pers Comm.,* Willoughby, 2023). This rise followed one of the most severe droughts on record, which forced animals to graze closer to concentrated water sources, increasing exposure to intermediate snail hosts, and was then followed and compounded by successive La Niña years that created warm, wet conditions favourable for snail survival and parasite transmission (Boray, 1964; Boray et al., 1969; Bureau of Meteorology, 2025; Neira et al., 2025). While environmental change likely contributed to this observed increase, many farmers suspected treatment failure due to drug resistance, prompting a local representative to contact researchers at the Sydney School of Veterinary Science (SSVS) at the University of Sydney, Australia in November 2023.

To confirm or deny these suspicions, we co-designed a diagnostic field investigation in collaboration with local livestock producers to determine the prevalence of *F. hepatica* and evaluate drug efficacy on affected farms in the NSW Southern Tablelands. By directly engaging with the most current guidelines by the World Association for the Advancement of Veterinary Parasitology (W.A.A.V.P.) on diagnosing anthelmintic resistance in ruminants, this study demonstrates both the utility and the limitations of applying standardised criteria to flukicide resistance in naturally infected livestock (Coles et al., 1992; Burden et al., 2024). In doing so, it builds on earlier Australian reports of TCBZ resistance, while providing insights into how these guidelines function in practice, particularly under the constraints of farmer-led, real-world investigations (Overend and Bowen, 1995; Brockwell et al., 2014; Elliott et al., 2015; George et al., 2019; Kelley et al., 2020).

Because livestock producers in *F. hepatica* endemic regions, such as the NSW Southern Tablelands, rarely manage liver fluke in isolation and increasingly rely on combination products targeting multiple parasite species (Leathwick and Besier, 2014; Playford et al., 2014; Preston et al., 2019), we also characterised the gastrointestinal nematode (GIN) communities co-infecting study animals using Nemabiome deep amplicon sequencing (Avramenko et al., 2015). This approach provided a broader perspective on parasite diversity and genomic signatures of anthelmintic resistance to Benzimidazoles (BZs) and Levamisole (LEV) in *Haemonchus contortus*, offering producers evidence-based insights into the wider challenges that accompany liver fluke control (Francis et al., 2024).

## 2 Materials and methods

### 2.1 Farm enrolment

A total of 20 farms (15 sheep, four cattle, one goat) with a history of *F. hepatica* infection were pre-screened after an information session was held locally in Gunning, NSW, in February 2024. Pre-screening involved farmers opportunistically collecting faecal samples for routine diagnostics (sedimentation/FEC and cELISA) over a period of four months. Samples were submitted to the SSVS at the University of Sydney, where they pooled per farm before being thoroughly mixed and 3 (sheep/goat) or 6 g (cattle) was taken for sedimentation/FEC and 0.5 g (sheep/goat) or 2 g (cattle) for cELISA (see section 2.3).

### 2.2 Drug efficacy field investigation

To investigate TCBZ resistance as the cause of drug failure, we followed the most recent W.A.A.V.P. guidelines for evaluating the efficacy of anthelmintics in ruminants as closely as possible (Kaplan et al., 2023; Burden et al., 2024). The study was conducted in July 2023, during the Austral winter. On Day 0, animals from properties included based on the pre-screening results were individually weighed and randomly allocated into three (sheep and goats) or two (cattle) treatment groups of 15 animals per group. The treatment groups included a negative control (water; H_2_O), TCBZ (Fasinex®), and a positive control (a combination product containing closantel at 50 g/L with abamectin at 1 g/L, AVOMEC Duel®; CLOS/AVM for sheep or albendazole, Alben®; ABZ for goats). No positive control was included on the cattle property. The combination product, AVOMEC Duel®, was selected as the positive control for sheep due to lack of availability of closantel as a single active for three months leading up to the trial commencement (referred to as CLOS/AVM hereafter). Although this product contains a higher dose of closantel than is recommended for use in sheep against *F. hepatica* (50 g/L vs. 37.5 g/L), it was considered appropriate in this instance because it is the drug of choice for use in sheep against drug resistant *H. contortus* and late immature *F. hepatica* in the region (*Pers Comm*., Willoughby, 2024).

Treatments were orally administered by farmers according to individual body weight at the recommended dosage as per the manufacturer's recommendations under guidance by researchers from The University of Sydney. On the Goat Property, both TCBZ and ABZ were administered at 1.5 × the sheep dose as recommended for use against *F. hepatica* in this species (*Pers Comm*., Elanco, 2024; Foreyt, 1988). At the time of drug administration, animals were observed to ensure treatments were swallowed, before being individually marked with stock spray for ease of identification and mustering 21 days later. Any animal that expelled the treatment was removed from the study and replaced to ensure accurate dosage. Animal weight, body condition score (BCS), gender, age, and previous liver fluke drug treatment history were recorded at the time of sample collection. BCS assessments were conducted by the same person for the duration of the trial to reduce variation. Scales and drench guns were calibrated by SSVS researchers at each property to ensure accuracy and consistency across visits. Fresh faecal samples were collected by the respective farmers *per rectum*, individually bagged, and stored at 4 °C until analysis at the SSVS, the University of Sydney. Animals were co-grazed for the duration of the study to reduce variation between treatment groups. After 21 days, the properties were visited for a second sample collection, during which time animals were individually weighed and scored again. All animal handling was conducted by the farmers as part of a routine diagnostic investigation with researchers from the SSVS present to ensure adherence to the W.A.A.V.P. guidelines. Existing ear-tags were numbered with permanent marker to facilitate animal-level identification on Day 21.

### 2.3 Diagnostics

#### 2.3.1 Sedimentation and FECs

To diagnose patent *F. hepatica* infections, a standard sedimentation and FEC method was followed (Happich and Boray, 1969). Briefly, individual faecal samples (3 and 6 g for sheep/goats and cattle, respectively) were mixed thoroughly with 10 mL of water and sieved through a course sieve (270 μM) into a 250 mL conical flask. The flask was topped with water and left to sediment for 3 min. The supernatant was aspirated and discarded before the sediment was washed into a clean 100 mL conical flask and left to sediment for another 3 min. After this second sedimentation, the supernatant was again discarded and the remaining sediment was washed into a 15 mL centrifuge tube, topped with distilled water and left to sediment for a final 3 min. The supernatant was aspirated, leaving 2 mL of sediment for analysis. To facilitate easier counting, two drops of methylene blue (1%) were added to each sediment. Sediments were rinsed into Perspex counting chambers (5 × 15 × 1 cm) and examined under an Olympus LG-PS2 stereomicroscope at 15 × magnification. Individual FECs were expressed as eggs per gram (EPG) of faeces for each animal, with counts for cattle divided by two (Happich and Boray, 1969). Once counted, sediments were collected into their original 15 mL centrifuge tube and stored at 4 °C. Day 21 samples were only counted if the corresponding mob met the study inclusion criteria (see Section 2.4.2), though all samples were processed and stored at 4 °C for consistency and later referral if necessary. To minimise variation between counts, Day 0 and Day 21 samples for each individual property were counted by the same person.

#### 2.3.2 Coproantigen ELISA

To diagnose prepatent *F. hepatica* infections and validate FEC-positive results, a commercially available cELISA was used on individual samples to detect *F. hepatica* antigen in faeces (Mezo et al., 2004). The test was performed as per manufacturer's instructions (BioX Diagnostics BIO K 201/2, batch number FASA24D29) by the same person to reduce variability with minor modifications as follows. Upon collection, faecal samples were stored at −20ᵒC in aliquots (0.5 g for sheep/goat; 2 g for cattle) for a maximum of 20 days. Samples were later thawed overnight in 2 mL dilution buffer on a slow rocking platform (Brockwell et al., 2013). The following day, aliquots were spun at 1000×*g* for 10 min and supernatants were collected and frozen until analysis (Brockwell et al., 2013). Five known negative *F. hepatica* samples, obtained from calves maintained on the University of Sydney's farm in Camden, NSW, and confirmed negative by routine sedimentation/FEC and a commercial serological ELISA conducted at the Elizabeth Macarthur Agricultural Institute (EMAI), were used as negative controls. For each treatment group per property, Day 0 and Day 21 samples were assayed on the same plate and run in technical duplicate to minimise variation. Optical Densities (OD) were read at 450 nm using a FLUOstar Omega plate reader (BMG Labtech) and the mean OD was used for interpretation of the results as per the manufacturer's instructions. Each run included a positive reference provided with the kit. As per the kit instructions, samples were considered positive if they had a Discrimination Factor (DF) ≥ 8%. Although alterations to the DF to increase diagnostic sensitivity were considered, they were ultimately not applied to align with commercial diagnostic laboratory methods and reflect the real-world scenario faced by producers attempting to diagnose drug resistance themselves (Brockwell et al., 2013; Calvani et al., 2017).

#### 2.3.3 DNA isolation and qPCR

To further confirm the FEC and cELISA results, a diagnostic qPCR assay specific to *F. hepatica* eggs was applied to five randomly selected samples from each treatment group on Day 0 and 21 (Calvani et al., 2017). Briefly, FEC sediments stored in 15 mL tubes were centrifuged at 2500×*g* for 10 min and supernatants were removed by aspiration with disposable 2 mL transfer pipettes. The tubes were cut open using a universal poly pipe cutter (Holman, Australia) to facilitate removal of the pellet. Total genomic DNA was isolated from the entire pellet using an Isolate II Fecal DNA Kit (BioLine, Australia) according to the manufacturer's instructions following mechanical disruption for 40 s at 6.0 m/s using a high-speed benchtop homogeniser (FastPrep-24, MP Biomedicals, Australia). A negative extraction control (ddH_2_O) was included in each extraction run to detect contamination. DNA was eluted into 100 μL elution buffer and stored at −20 °C prior to amplification.

A SYBR-chemistry real-time PCR assay detecting *F. hepatica* ITS2 rDNA from eggs was utilised as previously described (Calvani et al., 2017). Individual qPCR assays were performed at a final volume of 20 μL, containing SensiFAST™SYBR® No-ROX mix (Meridian Bioscience, Australia), and 2 μL of template DNA. Forward and reverse primers, SSCPFaF [S0754] and SSCPFaR [S0755], were included at a final concentration of 400 nM. Each qPCR assay was run in duplicate on a CFX96 Real-Time PCR Detection System (BioRad, Australia) under the following conditions: initial denaturation at 95 °C followed by 40 cycles of 95 °C for 5 s and 10 s at 60 °C, and a final melt curve analysis. All qPCR runs included a no template control (NTC; ddH_2_O).

#### 2.3.4 Nematode diagnostics

Concurrent GIN infections were determined by routine faecal flotation, FEC, Nemabiome and genomic resistance marker detection. Nematode FECs (henceforth referred to as nFECs) were performed in bulk per treatment group on samples collected on Day 0 and Day 21 for each farm using a highly sensitive Mini-FLOTAC technique as per the manufacturer's instructions (Cringoli et al., 2017). Larval cultures were performed in bulk per property if the pooled nFEC was ≥100 EPG according to a standard procedure outlined in the Australian Standard Diagnostic Techniques for Animal Diseases (ASDT) (Lyndal-Murphy, 1987).

A mixed metabarcoding deep amplicon sequencing workflow was applied to bulk Day 0 larval cultures from each property to simultaneously determine nematode species composition (ITS-2) and genomic resistance profiles to benzimidazoles (BZ; *β-tubulin isotype-1*) and levamisole (LEV; *acr-8 exon*, *H. contortus* only), as previously described (Francis et al., 2024). Larval cultures were centrifuged at 1000×*g* for 2 min and total genomic DNA was isolated from the resulting pellets using the Monarch Genomic DNA Purification kit (New England Biolabs, Australia). Extractions followed the manufacturer's instructions for tissue lysis, and a negative extraction control (ddH_2_O) was included in each batch to detect potential contamination. DNA was eluted into 100 μL of elution buffer (10 mM Tris-HCl, pH = 9.0, 0.1 mM EDTA) and stored at −20 °C.

Amplification assays were performed using Illumina adapter-linked primers (Avramenko et al., 2015, 2019; Francis et al., 2024). First-stage PCRs targeting LEV, BZ, and ITS-2 loci were performed individually using SYBR chemistry on a Myra robotic liquid handler (Bio Molecular Systems, Australia) in 35 μL reactions containing SensiFAST™SYBR® No-ROX mix (Meridian Bioscience, Australia), 2 μL of template DNA (neat for LEV/BZ, 1:1000 dilution for ITS-2), and primers at final concentrations of 400 nM. Cycling was carried out on a CFX96 Real-Time PCR Detection System (BioRad, Australia) with the following conditions: initial denaturation at 95 °C for 3 min, followed by 25–32 cycles of 95 °C for 5 s, 60 °C for 15 s, 72 °C for 15 s, and a final melt curve analysis. Each run included an NTC (ddH_2_O) to monitor for contamination. Amplicons were pooled in a 1:1:1 ratio and submitted to the Ramaciotti Centre for Genomics for sequencing using a 384-indexed amplicon format on an Illumina MiSeq platform.

Bioinformatic sequence analysis was performed in R (version 4.2.2), with LEV, BZ and ITS-2 sequences (paired FastQ files) processed independently using the ‘dada2’ pipeline (version 1.26.0) as previously described (Francis et al., 2024). Pipeline outputs were manually checked in CLC Main Workbench (version 22.0), and species/genotype proportions were calculated in Microsoft Excel (version 2207). Final outputs were visualised in GraphPad Prism (version 9.5.1) as stacked bar charts to represent species composition and resistance allele frequencies across properties.

#### 2.3.5 Identification of pseudo-parasites confounding morphological diagnosis

During routine sedimentation and FEC analysis of faecal samples, eggs with similar morphology to *F. hepatica* were observed in both pre-screening and study samples. This justified additional validation with an *F. hepatica*-specific diagnostic qPCR (Section 2.3.3 (Calvani et al., 2017);). On closer microscopic examination, the eggs appeared more consistent with those of free-living mites (Fig. 8D). To confirm their identity, DNA was extracted from >5 F. hepatica eggs and >5 suspected mite eggs from two pre-screening samples following the protocol described in Section 2.3.3.

The V9 hypervariable region of the 18S rRNA gene was subsequently amplified using Illumina adapter-linked primers S1148 and S1149 (Amaral-Zettler et al., 2009). PCRs were conducted in 20 μL volumes containing SensiFAST™ SYBR® No-ROX mix (Meridian Bioscience, Australia), 2 μL of template DNA, and primers at a final concentration of 400 nM. Cycling was performed on a CFX96 Real-Time PCR Detection System (BioRad, Australia) under the following conditions: initial denaturation at 95 °C for 3 min; 33 cycles of 95 °C for 5 s, 60 °C for 15 s, and 72 °C for 15 s; followed by a final melt curve analysis. A no-template control (ddH_2_O) was included to monitor for contamination. Amplicons were submitted to the Ramaciotti Centre for Genomics for sequencing in a 384-indexed amplicon format on an Illumina MiSeq platform. Bioinformatic sequence analysis was conducted in R (version 4.2.2), with paired-end FastQ files processed using the dada2 pipeline (version 1.26.0). Resulting amplicon sequence variants (ASVs) were manually inspected in CLC Main Workbench (version 22.0), and taxonomic assignments were performed using NCBI BLAST.

### 2.4 Statistical analysis

#### 2.4.1 Within-herd prevalence

The apparent and true within-herd prevalence of *F. hepatica* was reported for all mobs using Day 0 FECs. Apparent prevalence (*AP*) was calculated by taking the number of test-positive individuals divided by the total number of individuals tested (Rogan and Gladen, 1978). True prevalence (*TP*) utilises the diagnostic sensitivity (Se) and specificity (Sp) of a given test according to the method outlined by Rogan and Gladen (1978). The diagnostic Se and Sp of the traditional sedimentation after completion of the pre-patent period (eight weeks) were both 1 (Happich and Boray, 1969). For properties that met the TCBZ efficacy trial inclusion criteria (see Section 2.4.2), individual infection status on Day 0 and 21 was confirmed based on concordance between FEC, coproantigen ELISA, and qPCR results. For these properties, the assumed Se and Sp for the cELISA were 0.96 and 1, respectively (Mezo et al., 2004; Martinez-Sernandez et al., 2016).

#### 2.4.2 Selection of mobs for inclusion in the TCBZ efficacy field trial

According to the most recent W.A.A.V.P. guidelines for evaluating the efficacy of anthelmintics in ruminants, a minimum of six animals must be infected per group and the number of positive animals must be evenly distributed between treatment groups to ensure statistical power (Burden et al., 2024). To identify suitable mobs, individual animals were screened on Day 0 using a combination of sedimentation/FEC and cELISA. For each treatment group, a subset of five animals was also tested by qPCR to support infection confirmation. Animals were considered *F. hepatica*-positive on Day 0 for the purposes of inclusion in the trial if they were positive by FEC. Concordance with cELISA and qPCR results in the subset of tested animals supported classification of FEC results as true positives or negatives.

After calculation of within-herd prevalence, outliers were identified using the ROUT method (Q = 1%) and excluded from further analyses along with animals that returned negative FECs on Day 0. The results were assessed for normality using the Shapiro-Wilk test. The Shapiro-Wilk test displayed non-normality on log transformed data. For this reason, non-parametric statistical analyses were performed. Significant differences between treatment groups for properties were also assessed. The Kruskal-Wallis test was used to compare FEC results between the Day 0 treatment groups. Dunn's multiple comparisons test was used post hoc to identify significant differences between treatment groups on the two properties. Results were considered significant if P < 0.05. Data was analysed in GraphPad Prism version 10.3.1 and RStudio version 4.09.0–375.

#### 2.4.3 Determination of drug resistance

To determine drug efficacy and therefore confirm or deny suspicions of drug resistance, faecal egg count reduction tests (FECRT) and coproantigen reduction tests (CRT) were conducted on mobs that met the inclusion criteria. The addition of a negative control group allowed efficacy to be assessed using either paired (pre-vs post-treatment comparisons within a treatment group) or unpaired (comparison of post-treatment results vs negative control) data. This dual approach enhances interpretive power, particularly in mixed-age or low-burden infections, where individual variability might otherwise mask true reductions (Torgerson et al., 2014; Kaplan et al., 2023).

In line with current W.A.A.V.P. guidelines for diagnosing anthelmintic resistance in ruminants, reductions in egg counts were calculated using the online tool (eggCounts; http://shiny.math.uzh.ch/user/furrer/shinyas/shiny-eggCounts/) for animals that were FEC positive on Day 0 and available for follow up on Day 21 (Torgerson et al., 2014; Wang et al., 2018; Kaplan et al., 2023). This Bayesian hierarchical model supports both paired (individual-level) and unpaired (mob-level) FECRT analysis.

For the CRT, two methods were applied to the average DF for individual animals per property as described in Section 2.3.2 (Brockwell et al., 2014). Method 1 employs the RESO technique as recommended in the original W.A.A.V.P. guidelines, which compares post-treatment (Day 21) arithmetic means of treated and control groups (Coles et al., 1992). Method 2 takes the arithmetic means of the individual animal pre- (Day 0) and post-treatment (Day 21) cELISA result to derive individual CRs, which we then averaged (Coles et al., 1992). All FEC and cELISA data were managed in Microsoft Excel (version 16.9) and analysed using GraphPad Prism (version 10.3.1, GraphPad Software, USA).

## 3 Results

### 3.1 Pre-screening identified nine mobs for inclusion across eight farms

**Fig. 1:**
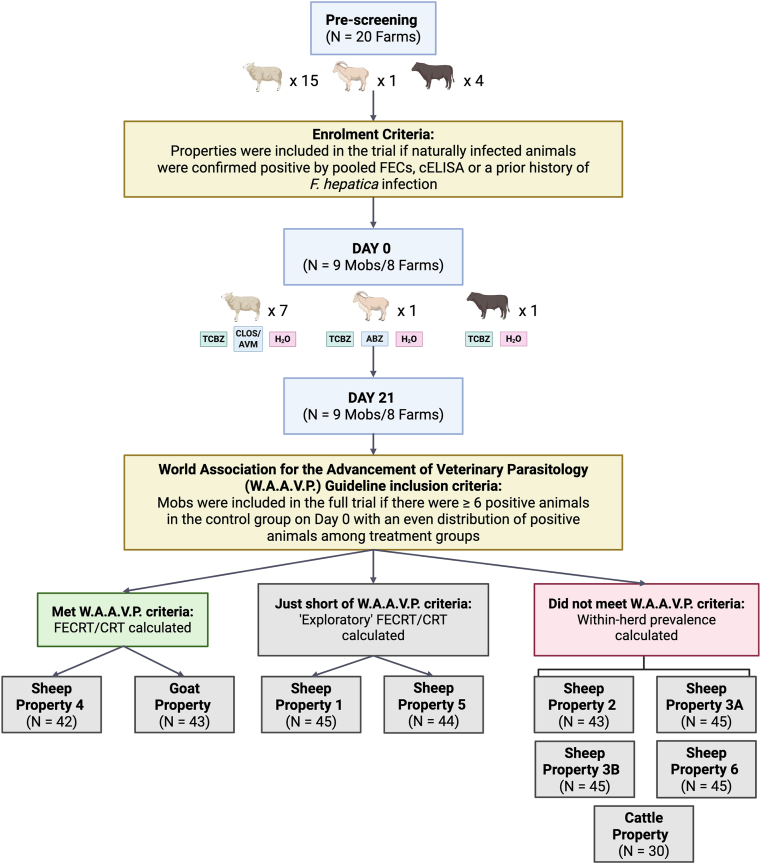
Inclusion criteria for a *Fasciola hepatica* triclabendazole (TCBZ) field efficacy study in the Southern Tablelands of New South Wales, Australia. Twenty farms (15 sheep, 1 goat, 4 cattle) were pre-screened for *F. hepatica* infection. Nine mobs from eight farms (seven sheep, one goat, one cattle) were included based on positive pooled diagnostics or a prior history of infection. On Day 0, individual faecal samples were collected and treatment groups were assigned (TCBZ, albendazole; ABZ [goats]/closantel and abamectin; CLOS/AVM [sheep], and untreated controls; H_2_O). Samples were collected 21 days later and faecal egg count reduction tests (FECRT) and coproantigen reduction tests (CRT) were conducted on mobs that met the W.A.A.V.P. criteria (≥6 positive control animals, even distribution between groups) on Day 0. ‘Exploratory’ analysis was conducted on mobs that were just short of the criteria and within-herd prevalence was calculated for those that did not meet the inclusion criteria. N = number of animals FEC tested on Day 0. Created using BioRender.com.

Of the 20 farms pre-screened, four were confirmed positive for *F. hepatica* via pooled diagnostic testing, while an additional four were included based on a history of liver fluke infection in the previous five years, as confirmed by the local rural supplies store manager (Supp. Fig. 1). From these eight properties (six sheep, one goat, one cattle), nine mobs were enrolled for Day 0 sample collection – including two from Sheep Property Three with different exposure histories (Sheep Property 3A and 3B; Fig. 1).

### 3.2 Within-herd prevalence of *F. hepatica* on selected properties in the NSW Southern Tablelands

In total, 382 animals were tested on Day 0, with an average number of 42 animals per mob (range: 30–45). The within-herd true prevalence of *F. hepatica* on Day 0 ranged from 0.00 to 74.42% (95% CI 0.00–0.00% to 61.38–87.46%; Table 1). Two mobs, (Sheep Property 3B and Six) were confirmed negative for *F. hepatica* infection by FEC, cELISA and qPCR (Supp. Fig. 4C and D). Although 4 animals from Sheep Property 3B recorded FECs of 1 EPG, subsequent qPCR testing confirmed that these were false positives due to morphological misidentification of eggs (Fig. 8; Supp. Fig. 4C; See Section 3.5).

**Table 1:** The true prevalence of *Fasciola hepatica* across eight farms and nine mobs in the New South Wales (NSW) Southern Tablelands. Prevalence was determined using a traditional sedimentation and faecal egg count (FEC). Two mobs enrolled from the same property (Sheep Property 3A and 3B) were included in the Day 0 sample collection due to different *F. hepatica* exposure histories.

Property	Animals FEC tested (Day 0)	Positive animals (Day 0)	True Prevalence (%) [95% CI]
**Sheep One**	45	14	31.11 [17.58–44.64]
**Sheep Two**	43	1	2.33 [0.00–6.83]
**Sheep 3A**	45	6	13.33 [3.40–23.27]
**Sheep 3B**	45	0	0.00 [0.00–0.00]
**Sheep Four**	42	31	73.81 [60.51–87.11]
**Sheep Five**	44	16	36.36 [22.15–50.58]
**Sheep Six**	45	0	0.00 [0.00–0.00]
**Goat**	43	32	74.42 [61.38–87.46]
**Cattle**	30	1	3.33 [0.00–9.76]

### 3.3 Inclusion criteria result in two fully and two partially eligible mobs

Only two mobs (Sheep Property Four and the Goat Property) met the full W.A.A.V.P. inclusion criteria for drug efficacy evaluation (Fig. 1). Two additional mobs (Sheep Property One and Five) narrowly missed these criteria but were included in the full study in an ‘exploratory’ capacity (Fig. 1). Samples and corresponding animal measurements (weight, BCS) were collected for all mobs on both Day 0 and Day 21. However, due to logistical constraints, post-treatment sedimentation/FEC and qPCR were only conducted for the four mobs that progressed to the full study (Sheep Properties One, Four, Five and the Goat Property, Supp. Fig. 2). cELISAs were performed on all Day 0 and Day 21 samples across the nine mobs.

**Fig. 2:**
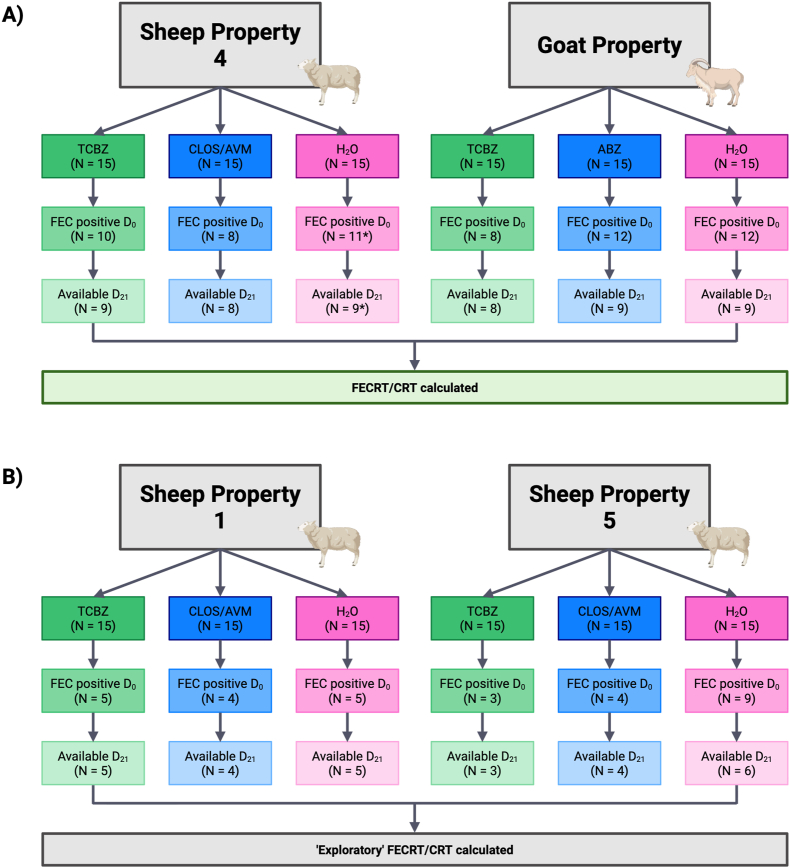
Summary of treatment group animals included in a *Fasciola hepatica* field efficacy study. (A) Sheep Property Four and the Goat Property met the full W.A.A.V.P. inclusion criteria (≥6 positive control animals, even distribution between groups) and were used to calculate faecal egg count reduction tests (FECRT) and coproantigen reduction tests (CRT). (B) Sheep Properties One and Five were retained for ‘exploratory’ efficacy analysis. Dark boxes indicate the number of animals included per treatment group on Day 0: triclabendazole (TCBZ); green, closantel/abamectin (CLOS/AVM, sheep) or albendazole (ABZ, goats); blue, and untreated controls (H_2_O); pink. Middle-tier boxes represent the number of FEC-positive animals on Day 0, and light boxes indicate the subset of these animals available for follow-up on Day 21. ∗Indicates the total animals available after two were removed as statistical outliers. Created using BioRender.com.

**Fig. 3:**
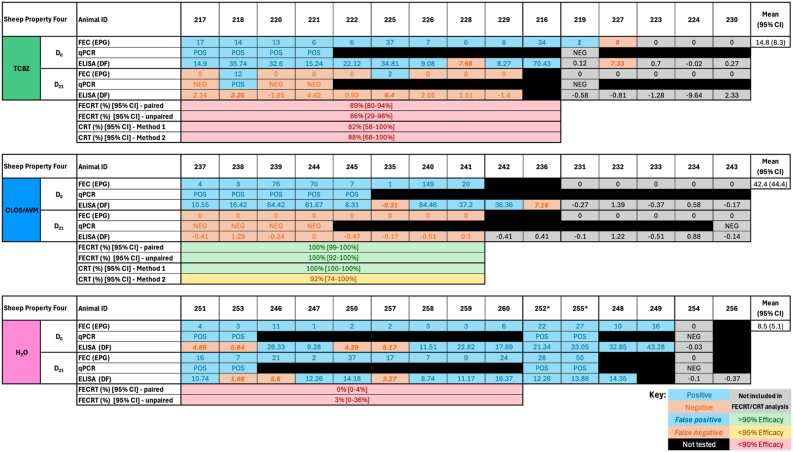
*Fasciola hepatica* drug efficacy on Sheep Property Four. Individual faecal egg counts (FEC) and coproantigen ELISA (cELISA) values are presented for animals by treatment group: triclabendazole (TCBZ); green, closantel/abamectin (CLOS/AVM); blue, and untreated controls (H_2_O); pink. Drug efficacy was assessed using faecal egg count reduction tests (FECRT) and coproantigen reduction tests (CRT), expressed as percentages. Two analyses were applied to each test (paired/unpaired or ‘Method 1/Method 2’). Samples included in efficacy analysis based on a positive FEC on Day 0 are highlighted in blue. Grey cells denote animals excluded from analysis due to negative Day 0 FECs. Negative results are shown in orange. A lack of concordance between testing methods indicating false positive (bold, italics, blue) or false negative (bold, italics, orange) results are shown. Outliers are indicated by stars above the animal ID. Black cells indicate animals unavailable for follow-up/insufficient sample volume on Day 21 or that were not selected for qPCR testing. Efficacy values are colour-coded: green for ≥95%, yellow for 90–94%, and red for <90%. Eggs per gram (EPG), Discrimination Factor (DF).

**Fig. 4:**
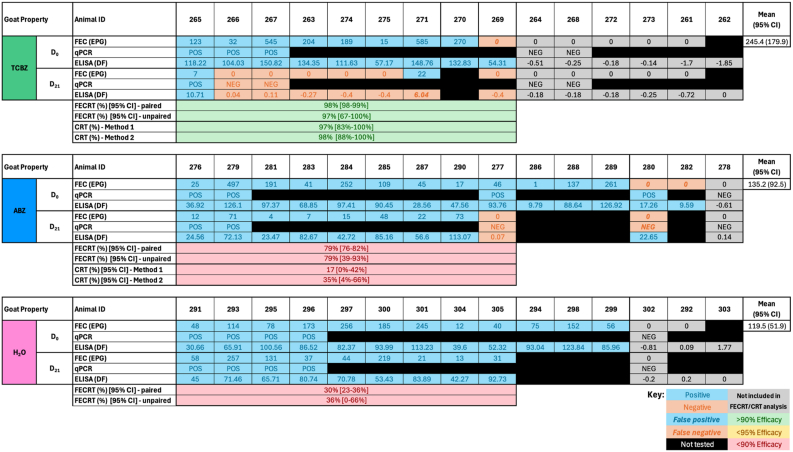
*Fasciola hepatica* drug efficacy on the Goat Property. Individual faecal egg counts (FEC) and coproantigen ELISA (cELISA) values are presented for animals by treatment group: triclabendazole (TCBZ); green, albendazole (ABZ); blue, and untreated controls (H_2_O); pink. Drug efficacy was assessed using faecal egg count reduction tests (FECRT) and coproantigen reduction tests (CRT), expressed as percentages. Two analyses were applied to each test (paired/unpaired or ‘Method 1/Method 2’). Samples included in efficacy analysis based on a positive FEC on Day 0 are highlighted in blue. Grey cells denote animals excluded from analysis due to negative Day 0 FECs. Negative results are shown in orange. A lack of concordance between testing methods indicating false positive (bold, italics, blue) or false negative (bold, italics, orange) results are shown. Outliers are indicated by stars above the animal ID. Black cells indicate animals unavailable for follow-up/insufficient sample volume on Day 21 or that were not selected for qPCR testing. Efficacy values are colour-coded: green for ≥95%, yellow for 90–94%, and red for <90%. Eggs per gram (EPG), Discrimination Factor (DF).

On Sheep Property Four, 10, 8, and 13 animals from the TCBZ, CLOS/AVM and H_2_O treatment groups, respectively, were FEC-positive on Day 0 (Fig. 2A). Two FEC-positive animals from the negative control group were excluded as statistical outliers (252 and 255), bringing the H_2_O treatment group down to 11 FEC-positive animals on Day 0, with 9 available and included in analysis on Day 21 (Fig. 2, Fig. 3). On the Goat Property, 8, 12 and 12 animals from the TCBZ, CLOS/AVM and H_2_O treatment groups, respectively, were FEC positive on Day 0, with 8, 9, and 9 included in the final analysis, respectively (Fig. 2, Fig. 4). FEC data on Sheep Property Four were not normally distributed in any treatment group (Shapiro-Wilk P < 0.05), while on the Goat Property, only the ABZ group showed non-normality (P = 0.0094). As a result, non-parametric tests were applied to both properties (Table 2, Table 3). The Kruskal-Wallis test showed significant overall variation on Sheep Property Four (P = 0.0404), though Dunn's post hoc comparisons did not reveal significant pairwise differences (Table 2). No significant differences were detected between treatment groups on the Goat Property (P = 0.3703, Table 3).

**Table 2:** Summary results on Day 0 on Sheep Property Four.

Sheep Property Four	TCBZ	CLOS/AVM	H_2_O	Total
Number of infected animals on Day 0	10	8	11	29
Day 0 FEC - Minimum	6	1	1	1
Day 0 FEC - Median	10.5	13.5	3	7
Day 0 FEC - Maximum	37	149	16	149
Day 0 FEC – Mean (95% CI)	14.80 (6.51–23.09)	41.25 (0.00–85.65)	5.55 (2.35–8.74)	18.59 (6.71–30.546)
Day 0 FEC - Std. Dev.	11.59	53.10	4.76	31.22
Kruskal-Wallis (P < 0.05)	Yes (P = 0.0404)			

(FEC; faecal egg count, TCBZ; triclabendazole, CLOS/AVM; closantel/abamectin).

**Table 3:** Summary results on Day 0 on the Goat Property.

Goat Property	TCBZ	ABZ	H_2_O	Total
Number of infected animals on Day 0	8	12	12	32
Day 0 FEC - Minimum	15	1	12	1
Day 0 FEC - Median	196.5	77.5	96	118.5
Day 0 FEC - Maximum	585	497	256	585
Day 0 FEC – Mean (95% CI)	245.40 (65.46–425.30)	135.20 (42.64–227.7)	119.50 (67.62–171.40)	156.80 (102.10–211.60)
Day 0 FEC - Std. Dev.	215.2	145.6	90.58	152.0
Kruskal-Wallis (P < 0.05)	No (P = 0.3703)			

(FEC; faecal egg count, TCBZ; triclabendazole, ABZ; albendazole).

**Fig. 5:**
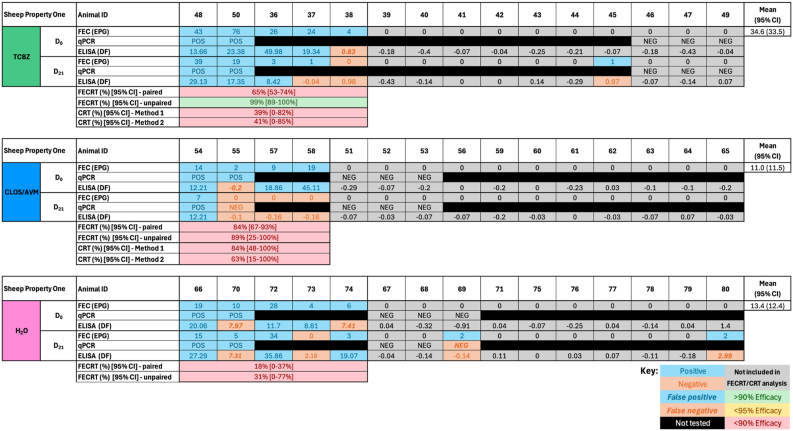
*Fasciola hepatica* exploratory drug efficacy on Sheep Property One. Individual faecal egg counts (FEC) and coproantigen ELISA (cELISA) values are presented for animals by treatment group: triclabendazole (TCBZ); green, closantel/abamectin (CLOS/AVM); blue, and untreated controls (H_2_O); pink. Drug efficacy was explored using faecal egg count reduction tests (FECRT) and coproantigen reduction tests (CRT), expressed as percentages. Two analyses were applied to each test (paired/unpaired or ‘Method 1/Method 2’). Samples included in efficacy analysis based on a positive FEC on Day 0 are highlighted in blue. Grey cells denote animals excluded from analysis due to negative Day 0 FECs. Negative results are shown in orange. A lack of concordance between testing methods indicating false positive (bold, italics, blue) or false negative (bold, italics, orange) results are shown. Outliers are indicated by stars above the animal ID. Black cells indicate animals unavailable for follow-up/insufficient sample volume on Day 21 or that were not selected for qPCR testing. Efficacy values are colour-coded: green for ≥95%, yellow for 90–94%, and red for <90%. Eggs per gram (EPG), Discrimination Factor (DF).

**Fig. 6:**
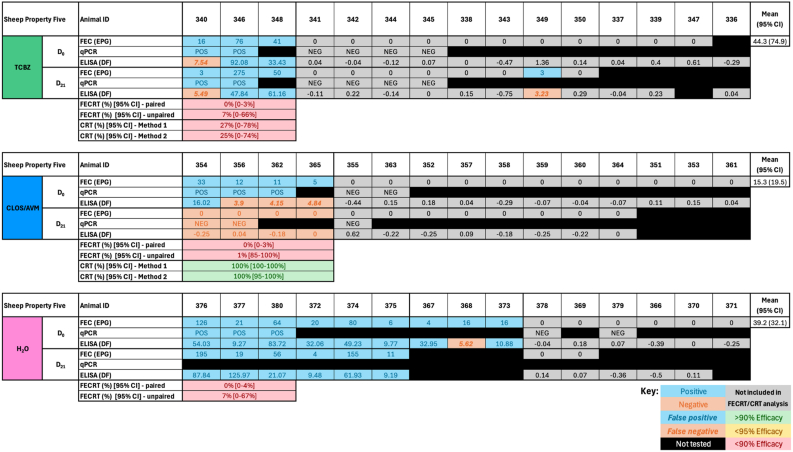
*Fasciola hepatica* exploratory drug efficacy on Sheep Property Five. Individual faecal egg counts (FEC) and coproantigen ELISA (cELISA) values are presented for animals by treatment group: triclabendazole (TCBZ); green, closantel/abamectin (CLOS/AVM); blue, and untreated controls (H_2_O); pink. Drug efficacy was explored using faecal egg count reduction tests (FECRT) and coproantigen reduction tests (CRT), expressed as percentages. Two analyses were applied to each test (paired/unpaired or ‘Method 1/Method 2’). Samples included in efficacy analysis based on a positive FEC on Day 0 are highlighted in blue. Grey cells denote animals excluded from analysis due to negative Day 0 FECs. Negative results are shown in orange. A lack of concordance between testing methods indicating false positive (bold, italics, blue) or false negative (bold, italics, orange) results are shown. Outliers are indicated by stars above the animal ID. Black cells indicate animals unavailable for follow-up/insufficient sample volume on Day 21 or that were not selected for qPCR testing. Efficacy values are colour-coded: green for ≥95%, yellow for 90–94%, and red for <90%. Eggs per gram (EPG), Discrimination Factor (DF).

Although Sheep Properties One and Five did not meet the W.A.A.V.P. inclusion criteria of six positive animals per treatment group on Day 0 (5, 4, 5 and 3, 4, 9 positive animals in the TCBZ, CLOS/AVM and H_2_O treatment groups, respectively; Fig. 2, Fig. 5, Fig. 6), both mobs were retained for ‘exploratory’ analysis based on sustained infection in multiple individuals through to Day 21 and relatively modest reductions in FECs.

A small number of animals enrolled on Day 0 were unavailable for follow up on Day 21. Reasons for lack of follow up included animals being missed during the second muster due to foggy winter conditions, absence of (or minimal) faeces at the time of sampling, and, as was primarily the case with the goats, missing or illegible individual IDs due to worn ear tags. Where <3 g faeces were collected, the cELISA was prioritised and no FEC was performed. These losses were accounted for by restricting efficacy calculations to animals with confirmed Day 0 infection and matching Day 21 samples.

### 3.4 Characterisation of co-infecting gastrointestinal nematodes and resistance profiles across trial properties

**Fig. 7:**
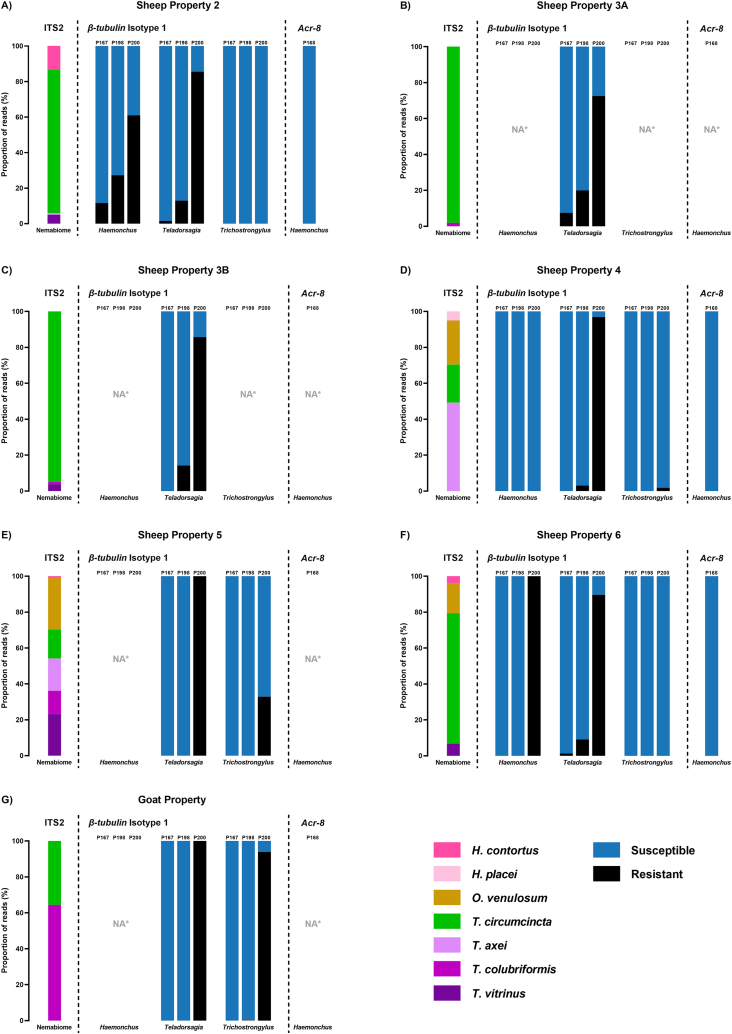
Nemabiome and anthelmintic resistance profiles of gastrointestinal nematodes from selected properties in the NSW Southern Tablelands. ITS2 deep amplicon sequencing (Nemabiome) was performed to identify nematode species composition within each mob. Concurrent next-generation sequencing of β-tubulin isotype-1 and acr-8 genes identified benzimidazole (BZ)- and levamisole (LEV)-resistant single nucleotide polymorphisms (SNPs), respectively. Bars represent the proportion (%) of sequencing reads for each nematode species (ITS2), and resistant versus susceptible alleles at the three major BZ resistance-associated SNP sites (codons 167, 198, 200), and the LEV resistance-associated SNP (codon 168, S168T). "NA∗" indicates the species was absent or there was insufficient read depth for reliable SNP determination due to the species comprising of <5% of the total Nemabiome.

Nematode FECs were evenly distributed between treatment groups on all properties on both sample dates, except for the CLOS/AVM group on Sheep Property Four, which saw a decline in EPG from 290 to 0 (Supp. Fig. 3). Sheep Property One and the Cattle Property recorded very low nFECs (<10 EPG), insufficient for successful larval culture, and were therefore excluded from further molecular analysis. Seven GIN species were identified in the remaining properties: *H*. *contortus*, *Haemonchus placei*, *Oesophagostomum venulosum*, *Teladorsagia circumcincta*, *Trichostrongylus axei*, *Trichostrongylus colubriformis*, and *Trichostrongylus vitrinus*, each matching the local ITS-2 reference database with over 98% identity. *T. circumcincta* was the dominant species on Sheep Properties Two, 3A, 3B, and Six, comprising 81%, 98%, 95%, and 72% of the Nemabiome, respectively (Fig. 7). *T. colubriformis* predominated at the Goat Property, accounting for 65% of the species composition. The remaining properties (Sheep Properties Four and Five) displayed more even and diverse nematode communities (Fig. 7).

**Fig. 8:**
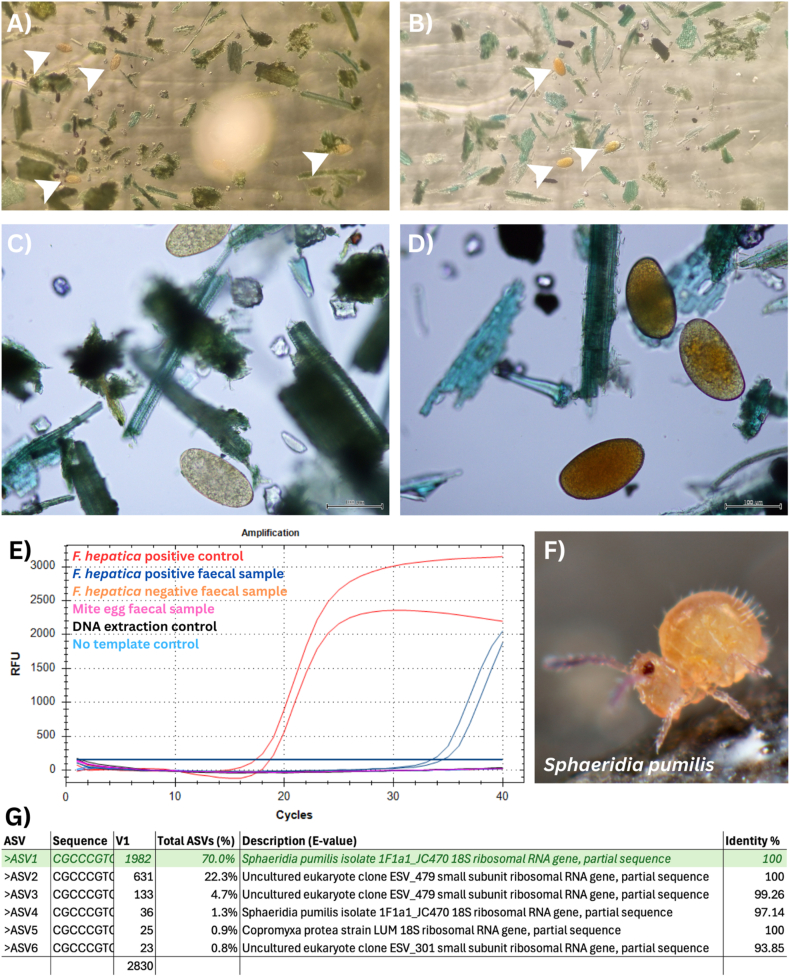
Morphological and molecular comparison of *Fasciola hepatica* eggs and morphologically similar mite eggs detected during faecal sedimentation. (A) *F. hepatica* eggs visualised at low power under a stereo microscope in a known positive faecal sedimentation sample (white arrows). (B) Mite eggs with comparable morphology observed during faecal egg counting (white arrows). (C) and (D) show the same *F. hepatica* (A) and mite (B) eggs, respectively, at higher magnification using a compound light microscope (scale bar = 100 μm). (E) *F. hepatica*-specific qPCR targeting the ITS-2 region of the rDNA confirm the eggs are not *F. hepatica* (positive control; red, *F. hepatica* positive faecal sample; dark blue, mite eggs; pink, *F. hepatica*-negative faecal sample; orange, DNA extraction control; black, no-template control (NTC); light blue. (F) Further investigation revealed their identity to mite eggs from the species *Sphaeridia pumilis* (source: Wikimedia Commons). (G) Amplicon be free-living oribatid deep sequencing confirmed 70% ASVs returned 100% species identity with *S. pumilis* 18S rDNA.

Resistance alleles were characterised in *Haemonchus*, *Teladorsagia*, and *Trichostrongylus* using deep amplicon sequencing of the *β-tubulin* isotype-1 locus, and in *Haemonchus* only for the *acr-8* locus, as this LEV resistance marker has not been validated in other genera (Fig. 7). BZ-resistant genotypes were widespread. The F200Y allele in *Teladorsagia* spp. was consistently high across all properties (mean = 90.1%, SD ± 0.9%), while F167Y and E198L were minimal (<8%). In *Trichostrongylus* spp., F200Y was the only resistance-associated SNP detected, generally absent to moderate (range = 0–33%), but reaching 94% at the Goat Property. In *Haemonchus* spp., resistance profiles varied markedly: all SNPs were absent on Sheep Property Four, F200Y was fixed at 100% on Sheep Property Six, and moderate frequencies of all three SNPs (F167Y, E198A, F200Y) were detected on Sheep Property Two (12%, 27%, 61%, respectively). In contrast, the LEV-resistant S168T variant in *Haemonchus* spp. was not detected on any of the three properties with sufficient sequencing coverage (Fig. 7).

### 3.5 Morphological similarity of free-living mite eggs confounds *F. hepatica* diagnosis by sedimentation

Sequencing of the amplicons from the suspected mite eggs yielded 2830 reads, which clustered into six ASVs. The majority of reads (70.0%) were assigned to *Sphaeridia pumilis* isolate F1fa1_JC470 with 100% identity, indicating this species was the dominant taxon detected (Fig. 8G). The remaining ASVs corresponded to uncultured eukaryote clones or other minor taxa at low abundance.

### 3.6 Triclabendazole resistance was detected on two properties in the NSW Southern Tablelands

#### 3.6.1 FECRT

FECRTs were conducted on four mobs; Sheep Properties One, Four, Five and the Goat Property. On Sheep Property Four and the Goat Property, which met the full inclusion criteria, 10, 8 and 11 animals (Sheep Property Four) and 8, 12 and 12 animals (Goat Property) tested FEC-positive in the TCBZ, CLOS/AVM/ABZ and H_2_O treatment groups on Day 0, respectively (Fig. 2, Fig. 3, Fig. 4). The average FEC burden in infected animals on Day 0 was relatively low on Sheep Property Four, at 14.8 ± 8.3 EPG (TCBZ), 42.4 ± 44.4 EPG (CLOS/AVM) and 8.5 ± 5.1 EPG (H_2_O) (Fig. 3). In contrast, the Goat Property exhibited substantially higher FECs, with average burdens of 245.4 ± 179.9 EPG (TCBZ), 135.2 ± 92.5 EPG (ABZ) and 119.5 ± 51.9 EPG (H_2_O) (Fig. 4).

Loss of efficacy occurs when there is percentage reduction in FECs of <90% (Burden et al., 2024). Two methods were used to calculate FECR as described in Section 2.4.3, using either paired (pre- and post-treatment samples from the same animals) or unpaired (treated vs. control animals) data. According to the paired analysis, the FECR of animals treated with TCBZ on Sheep Property Four was 89% [95% CI 80–94%], consistent with TCBZ resistance. Using the unpaired approach, the FECR was 86% [95% CI 28–98%] (Fig. 3). The wider confidence interval using this method reflects greater variability and uncertainty, highlighting the improved precision gained by using paired data. Both methods showed FECR of 100% for the CLOS/AVM treatment group, confirming susceptibility to this product.

In contrast, animals treated with TCBZ on the Goat Property demonstrated FECRs exceeding 95% using both paired and unpaired analyses, confirming TCBZ susceptibility in this mob (Fig. 4). Conversely, ABZ efficacy was notably reduced, with a paired FECR of just 79% [95% CI: 76–82%] and an unpaired FECR of 79% [95% CI: 29–93%] (Fig. 4). It is important to note that this flukicide has reduced efficacy against immature fluke (<12 weeks old) (Fairweather and Boray, 1999; Fairweather et al., 2020).

On Sheep Properties One and Five, FECRTs were conducted in an ‘exploratory’ capacity as these mobs narrowly missed the W.A.A.V.P. inclusion criteria, primarily due to insufficient numbers of infected animals in one or more treatment groups. On Sheep Property One, 5 animals in each of the TCBZ and H_2_O groups, and 4 in the CLOS/AVM treatment group, were FEC-positive on Day 0. Mean FECs in infected animals on Day 0 were 34.6 ± 33.5 EPG (TCBZ), 11 ± 11.5 EPG (CLOS/AVM) and 13.4 ± 12.4 EPG (H_2_O). Using paired data, the FECRT for TCBZ was 65% (95% CI: 53–74%), suggesting substantially reduced efficacy. The unpaired FECRT, however, returned a value of 99% (95% CI: 89–100%). This discrepancy likely reflects the low FECs in the untreated control group and therefore should be interpreted with caution. Notably, the latest W.A.A.V.P. guidelines indicate that a minimum sample size of five infected animals per group is required to achieve ≥80% statistical power (Kaplan et al., 2023). For the CLOS/AVM treatment group, paired and unpaired FECRTs returned values of 84% (95% CI: 67–93%) and 89% (95% CI: 25–100%), respectively. Collectively, these results strongly suggest the presence of TCBZ resistance in this mob but further testing with increased sample sizes is recommended before drawing definitive conclusions regarding CLOS/AVM efficacy (Fig. 5).

On Sheep Property Five, 3, 4 and 9 animals were FEC-positive on Day 0 in the TCBZ, CLOS/AVM and H_2_O treatment groups, respectively. The mean FEC on Day 0 was low across all groups, with average EPGs of 44.3 ± 74.9 (TCBZ), 15.3 ± 19.5 (CLOS/AVM) and 39.2 ± 32.1 (H_2_O). FECRTs for the TCBZ and CLOS/AVM treatment groups were very low (<10%) irrespective of calculation method (paired or unpaired), suggesting a substantial loss of efficacy for both products (Fig. 6). However, these findings should be interpreted with caution given the small sample sizes, low baseline egg counts, and considerable variability observed.

#### 3.6.2 CRT

CRTs were conducted on Sheep Properties One, Four, and Five, as well as the Goat Property, to complement the FECRT results. On Sheep Property Four, which fully met the W.A.A.V.P. inclusion criteria, cELISA testing identified 9, 8, and 9 positive animals in the TCBZ, CLOS/AVM, and H_2_O groups on Day 0, respectively (Fig. 3). Based on parallel FEC and qPCR results, 1, 1, and 4 animals in the respective groups were identified as false negatives when using the manufacturer's recommended cutoff (DF < 8). The CRT indicated a reduction in efficacy consistent with TCBZ resistance, as the results for both methods fell below the expected threshold for full efficacy (≥95%), with a coproantigen reduction (CR) of 82% [95% CI: 58–100%] using Method 1, and 88% [95% CI: 68–100%] with Method 2. These findings align with the FECRT results and support a diagnosis of TCBZ resistance on this property. In contrast, CLOS/AVM-treated animals showed high efficacy, with CRs of 100% [95% CI: 99–100%] (Method 1) and 100% [95% CI: 92–100%] (Method 2), consistent with the observed FECRT outcomes.

On the Goat Property, CRT results corroborated the FECRT findings, clearly distinguishing TCBZ susceptibility and ABZ resistance. On Day 0, 9, 14 and 12 animals in each treatment group (TCBZ, ABZ, and H_2_O) were positive for *F. hepatica* faecal antigens. No animals were considered false positives by cELISA on this property, likely due to the significantly higher burden compared to the Sheep Properties. High CRs were observed in the TCBZ group using both Method 1 (97% [95% CI: 83–100%]) and Method 2 (98% [95% CI: 88–100%]), consistent with the FECRT results. In contrast, CRs for ABZ-treated animals were well below the thresholds for efficacy: 17% [95% CI: 0–42%] with Method 1 and 35% [95% CI: 4–66%] with Method 2, confirming ABZ resistance on this property (Fig. 4).

On Sheep Property One, which was included in an ‘exploratory’ capacity, CRT results were highly variable and should be interpreted with caution. Four animals in the TCBZ-treated group were positive by cELISA on Day 0, with one additional animal considered a false negative based on a corresponding low but detectable FEC (4 EPG; Fig. 5). Method 1 yielded a CR of 39% [95% CI: 0–82%], and Method 2 returned a CR of 41% [95% CI: 0–85%], both of which fall well below the W.A.A.V.P. efficacy threshold, suggesting reduced TCBZ effectiveness in this group. Among CLOS/AVM-treated animals, 3 were cELISA-positive on Day 0, and an additional false negative (sample 55) was identified based on corroborating diagnostic data (Fig. 5). Method 1 yielded a CR of 84% [95% CI: 48–100%], while Method 2 indicated a lower CR of 63% [95% CI: 15–100%], with wide confidence intervals highlighting uncertainty around efficacy calculations in this mob.

On Sheep Property Five, CRTs demonstrated the variability of the cELISA in mobs with low burdens when used according to the manufacturer's recommendations. In the TCBZ treatment group, the low CRs (27% [95% CI: 0–78%]; Method 1 and 25% [95% CI: 0–74%]; Method 2), agree with the FECRT results. This is likely due to the low number of false negatives in this group on Day 0 (Fig. 6). In comparison, three of the four FEC positive animals in the CLOS/AVM treatment group returned false negative results on Day 0. Consequently, the CRT for this group was 100% [95% CI: 100-100%] (Method 1) and 100% [95% CI: 95–100%] (Method 2; Fig. 6). As with Sheep Property One, these results suggest loss of efficacy, but interpretation remains limited by small sample sizes and low pre-treatment burdens.

### 3.7 No significant production impacts were observed between treatment groups across the trial period

There were no statistically significant differences in BCS or weight between treatment groups on either Day 0 or Day 21 of the trial. However, a decline in BCS was observed in animals across all treatment groups and properties over the course of the trial. This trend was consistent throughout the region, which experienced an extreme cold snap during the trial period (data not shown).

## 4 Discussion

This study was designed to move beyond anecdote and systematically investigate suspected TCBZ resistance in the NSW Southern Tablelands, where farmers had reported a sharp increase in liver fluke burdens. By working in partnership with livestock producers and aligning the study with W.A.A.V.P. guidelines (Kaplan et al., 2023; Burden et al., 2024), we were able to both confirm TCBZ resistance on one sheep property (Sheep Property Four) and identify additional properties with potential loss of TCBZ efficacy (Sheep Property One and Five). Notably, the inclusion of ABZ as a positive control on the Goat Property revealed reduced efficacy representing, to our knowledge, the first potential report of ABZ resistance in *F. hepatica* infecting goats worldwide. This outcome is likely a reflection of sustained underdosing, as goats require 1.5 × the sheep dose due to their faster drug metabolism (Foreyt, 1988), yet discussions with producers indicated routine dosing at just 1.2 × the sheep dose. It should be noted that this product has reduced efficacy against immature *F. hepatica* (<12 weeks old) and hence further confirmation via a controlled efficacy test (CET) is recommended.

In our study, the combined use of sedimentation/FEC, cELISA, and qPCR was essential for detecting resistance and differentiating true from false negatives. We deliberately chose to present data from multiple diagnostic methods at the individual animal level instead of treatment-level averages to demonstrate the interpretive complexity that arises in low-burden mobs. This was particularly evident on Sheep Properties One, Four and Five, where several animals tested negative by cELISA using the recommended cutoff despite having detectable eggs and/or qPCR-confirmed infections. Decreased diagnostic sensitivity of the cELISA in naturally infected animals is a known limitation, particularly in immature or low burden infections (Brockwell et al., 2013; Calvani et al., 2017; George et al., 2017, 2019). Rather than adjusting the cELISA threshold *post hoc* to increase the diagnostic sensitivity of this method, we chose to retain it to reflect the diagnostic conditions typically applied in commercial laboratory settings, which producers and veterinarians rely on for drench test results to determine treatment success. Accurate diagnosis was further compounded by the detection of morphologically similar eggs in several samples, which, under low-power microscopy, looked like *F. hepatica* and could have been mistakenly recorded as positive in routine sedimentation tests (Fig. 8A and B). Further investigation identified these eggs as free-living oribatid mites of the genus *Sphaeridia*. Their persistence post-treatment, even at low levels, may lead to false conclusions of treatment failure in commercial diagnostic settings and could have contributed to the suspicions of drug resistance in the lead up to the current study. In the absence of species confirmation using molecular methods, commercial laboratories relying solely on egg counts or cELISAs may risk misdiagnosing drug resistance, underscoring the need for consistent diagnostic workflows and consideration of confirmatory testing in cases of apparent treatment failure.

While producer suspicions were, in some cases, validated by evidence of drug resistance, other instances highlighted the challenges associated with conducting drug resistance trials in naturally infected, low-burden field populations. Further, our findings demonstrate that “drug failure” can arise through multiple causes, and suspicions should always be verified. Specifically, three factors likely contributed to the reported 230% increase in liver fluke burden in the region: (1) climate variability, such as drought followed by successive La Niña years contributing to increased interaction between livestock and intermediate snail hosts in permissible habitats; (2) pseudo-parasites, such as *S. pumilis* eggs that are morphologically similar to *F. hepatica* eggs under low-power microscopy, potentially leading to false positive FECs due to reduced specificity; and (3) diagnostic sensitivity issues in low-burden or immature infections, particularly false negatives associated with the commercial cELISA (Calvani et al., 2017; George et al., 2017). These confounders illustrate how diagnostic and environmental challenges can obscure the diagnosis of anthelmintic resistance in the field. It is worth noting that large numbers of lymnaeid snails were observed during the study period, which occurred in the middle of the Austral winter when temperatures were regularly <7 °C during the day. The occurrence of intermediate snail hosts outside their seasonal window in prior years may have contributed to the reported increase in *F. hepatica* infections and is worth investigating further given that other studies have recently noted altered transmission dynamics due to climate variability and invasive intermediate hosts (Neira et al., 2025).

Interpretation of flukicide efficacy is highly dependent on the analysis approach, particularly in field-based studies. To interpret the FECRTs, we conducted both paired and unpaired analyses. While unpaired analyses remain common in field settings, the guidelines now emphasise the superiority of paired designs where feasible, as they account for individual variation in baseline egg counts and yield conclusive results more often than unpaired methods (Kaplan et al., 2023). This distinction was particularly relevant on Sheep Property Four, where paired FECRT analysis demonstrated an 89% (95% CI: 80–94%) reduction following TCBZ treatment compared to an 86% (95% CI: 29–98%) reduction using the unpaired approach, both just below the 90% threshold for efficacy. Although numerically close, the wider confidence interval of the unpaired analysis reflects lower statistical certainty and reduced power, underscoring the risk of misclassification in low-burden settings. Two animals on Sheep Property Four remained positive on Day 21 with relatively minor reductions in FEC, materially affecting the group-level estimates and supporting a classification of confirmed TCBZ resistance (Fig. 3). In contrast, on the Goat Property, paired and unpaired FECRTs showed similarly high reductions (98% and 97%, respectively), despite two animals also remaining positive on Day 21. While the uncertainty interval (95% CI: 98–99% and 67–100%, respectively) of the unpaired method remained low in this case, the substantial group-level reductions outweighed the impact of the residual positives, supporting a conclusion of susceptibility (Fig. 4). This comparison between Sheep Property Four and the Goat Property illustrates that post-treatment positives must be interpreted within the context of overall burden, reduction magnitude, and the certainty provided by the confidence intervals. It also highlights the critical importance of rigorous study design and cautious interpretation when reductions and confidence intervals hover close to efficacy thresholds, as inconclusive results remain a recognised and important outcome category under the current W.A.A.V.P. guidelines (Kaplan et al., 2023).

In parallel with *F. hepatica*, we also characterised the GIN communities present across the study properties to assess potential confounding parasite burdens and explore broader patterns of anthelmintic resistance in the region. In practical farm settings where *F. hepatica* is endemic, nematodes and liver fluke are co-managed, often using overlapping control strategies, and failures in either system can exacerbate parasite impacts on animal health and productivity (Sargison, 2011). Additionally, the Australian pharmaceutical industry, and as a result, livestock producers, are increasingly turning to multi-active products, further complicating anthelmintic resistance management (Leathwick and Besier, 2014; Lamb et al., 2017; Kotze and Hunt, 2023). ITS-2 nemabiome sequencing identified seven nematode species across sheep and goat mobs, with *T. circumcincta* dominating most sheep farms and *T. colubriformis* predominating on the Goat Property (Fig. 7). BZ resistance-associated SNPs were widespread, with the F200Y mutation in β-tubulin detected at 100% frequency on multiple properties, confirming the high level of anthelmintic resistance in the region. Complementing traditional liver fluke resistance trials with molecular surveillance for GIN resistance offers farmers a more comprehensive, evidence-based approach to managing both parasite groups simultaneously. The ability to detect species-specific burdens and resistance profiles within a single sampling workflow enables more strategic, targeted treatment decisions and supports the adoption of integrated parasite management practices. In turn, this may reduce unnecessary anthelmintic treatments, slow the development of resistance, and improve long-term farm productivity. However, further work is required to standardise and validate molecular assays for anthelmintic resistance detection across different GIN species, including the correlation of SNP markers with phenotypic resistance outcomes and the establishment of predictive thresholds for field use (Antonopoulos et al., 2024; Šlapeta et al., 2024). Additionally, newer technologies that increase sample throughput and subsequently reduce result turnaround time require validation and development (Antonopoulos et al., 2024). Ultimately, the routine application of molecular diagnostics is likely to play a critical role in preserving susceptible refugia, a cornerstone of sustainable parasite control, by identifying resistance early and informing treatment strategies that protect susceptible parasite populations through targeted selective treatment (Hodgkinson et al., 2019).

Looking ahead, the development of molecular markers for drug resistance in *F. hepatica*, particularly against TCBZ, holds promise. Recent work has identified β-tubulin isotype 2 polymorphisms and other candidate loci associated with reduced TCBZ efficacy, offering the potential for non-invasive, genomic resistance diagnostics (Beesley et al., 2023). When integrated with existing molecular tools for GINs, this could allow for comprehensive, species-specific resistance profiling from a single faecal sample, providing producers with a practical ‘one-stop shop’ for parasite surveillance and treatment planning. However, studies have also revealed that TCBZ resistance likely evolves independently across regions, with distinct selection signatures in the United Kingdom versus South America (Choi et al., 2025). This geographic heterogeneity underscores the need for regionally validated molecular makers to support the development of locally appropriate molecular workflows. Crucially, these tools must be benchmarked against phenotypic assays such as the FECRT and CRT to ensure diagnostic reliability under field conditions. With appropriate validation, combined molecular surveillance of livestock nematodes and trematodes has the potential to reduce reliance on *in vivo* efficacy trials, enabling earlier detection of emerging resistance while helping to preserve refugia and slow the spread of resistant alleles (Hodgkinson et al., 2019; Antonopoulos et al., 2024; Šlapeta et al., 2024).

While multiple studies have sought to diagnose TCBZ resistance in *F. hepatica* in Australia, none have met all W.A.A.V.P. field efficacy guidelines. Earlier reports in Australia, including the foundational work by Overend and Bowen (1995), and subsequent trials by Brockwell et al. (2014), Elliott et al. (2015), and Kelley et al. (2020), identified resistance through FECRT and CRT, but varied in their design, sample sizes, inclusion of control groups, and diagnostic approaches (Supp. Fig. 5). In the present study, we aimed to align with these guidelines as closely as possible but faced several practical challenges. Specifically, logistical constraints prevented us from (1) conducting individual pre-treatment FECs for animal-level inclusion, (2) sampling animals over three consecutive days, and (3) fully blocking treatment groups within mobs. These limitations are not uncommon in field-based studies with naturally infected livestock and underscore the need for more flexible, species-specific protocols. We propose that the development of W.A.A.V.P. guidelines tailored to *F. hepatica* is now warranted. Given its complex life cycle, distinctive biology, and the increasing reliance on cELISA and emergence of molecular diagnostic methods, *F. hepatica* presents unique challenges not adequately captured in guidelines primarily designed for the diagnosis of anthelmintic resistance in GINs. Such bespoke recommendations considering these challenges would facilitate higher-quality resistance surveillance and study design, particularly in mixed-age, low-burden, and multi-parasite field settings where efficacy thresholds are often difficult to interpret with confidence.

Ultimately, this study moves beyond suspicion to deliver a farmer-led systematic investigation of suspected flukicide failure on naturally infected farms in the NSW Southern Tablelands, confirming TCBZ resistance on one sheep property, potential resistance on two others, and the first potential case of ABZ resistance in *F. hepatica* naturally infecting goats globally. By aligning with the updated W.A.A.V.P. guidelines and employing a multi-modal diagnostic approach, we were able to characterise divergent patterns of anthelmintic resistance across both liver fluke and gastrointestinal nematodes, highlighting the need for refined diagnostic methods for economically important livestock parasites. These findings demonstrate the practical utility of integrated faecal diagnostics for guiding targeted treatment decisions in field settings, while also underscoring the limitations of current diagnostic methods in low-prevalence or burden field populations. As anthelmintic resistance continues to spread, there is an urgent need for *Fasciola*-specific W.A.A.V.P. guidelines and validated molecular workflows that enable earlier, more accurate detection of emerging resistance and support the long-term preservation of anthelmintic efficacy through targeted-selective-treatment and refugia-conscious management.

## CRediT authorship contribution statement

**Chelsie Uthayakumar:** Writing – original draft, Visualization, Methodology, Investigation, Formal analysis, Data curation, Conceptualization. **Hayley Martinez DeCristi:** Writing – original draft, Visualization, Methodology, Investigation, Formal analysis, Data curation. **Emily Kate Francis:** Writing – review & editing, Writing – original draft, Visualization, Validation, Methodology, Investigation, Formal analysis, Data curation. **Roger Alan Willoughby:** Project administration, Conceptualization. **Shannon Taylor:** Writing – review & editing, Software, Resources, Methodology, Formal analysis, Data curation. **Nichola Eliza Davies Calvani:** Writing – review & editing, Writing – original draft, Visualization, Supervision, Resources, Project administration, Methodology, Investigation, Funding acquisition, Formal analysis, Data curation, Conceptualization.

## Funding

N.E.D.C. is an Australian Research Council (ARC)-funded Discovery Early Career Researcher (DECRA) Fellow (DE240100295). This work was completed in partial fulfillment for the requirements of the Animal and Veterinary Bioscience (AVBS) Honours Degree, 10.13039/501100001774The University of Sydney (C.U.) and funded by the 10.13039/501100022418SSVS Late Dorothy Minchin Bequest. C.U. was the recipient of an Australian Wool Education Trust (AWET) scholarship from 10.13039/100008708Australian Wool Innovation (10.13039/100008708AWI). C.U., R.A.W. and N.E.D.C. designed the project; C.U., H.D.C., E.K.F. and N.E.D.C. undertook the methodology and data acquisition; C.U., H.D.C., S.T., E.K.F. and N.E.D.C. conducted the data analysis, curation and visualisation. The original draft of the manuscript was written by C.U. and N.E.D.C. All authors contributed to the final version of the manuscript.

## Declaration of conflict of interest

R.W. owns and runs Gunning Ag & Water Solutions, from where he has provided advice to farmers in the region on livestock parasite control for >30 years. Through his parasite surveillance as part of his interest in regional livestock health via routine recording keeping, he noticed an increase in *F. hepatica* in the region in 2019/2020 and suspected drug resistance as a cause of drug failure. After contacting N.E.D.C. at the University of Sydney, he coordinated the trial with local producers but had no financial involvement in its design or outcomes. He remains committed to evidence-based best practice for the health and wellbeing of livestock and producers in the region.

## Figures and Tables

**Figure:**
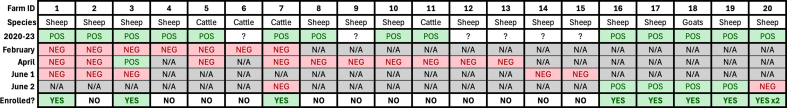


**Figure:**
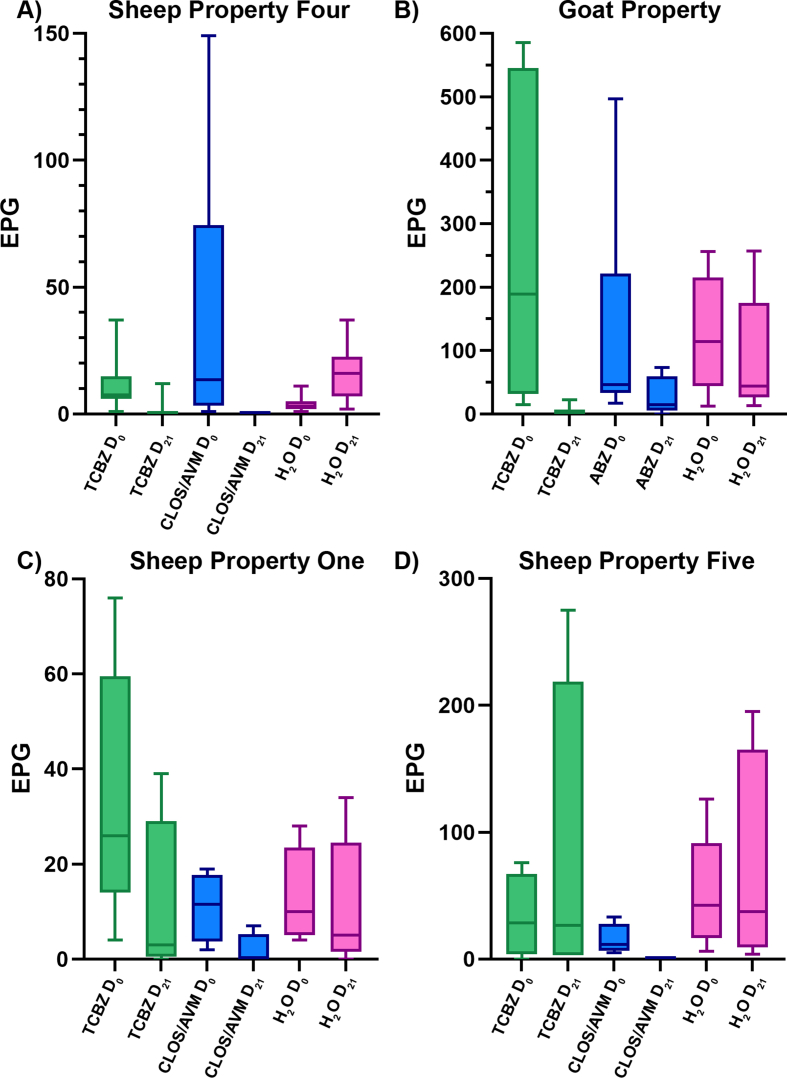


**Figure:**
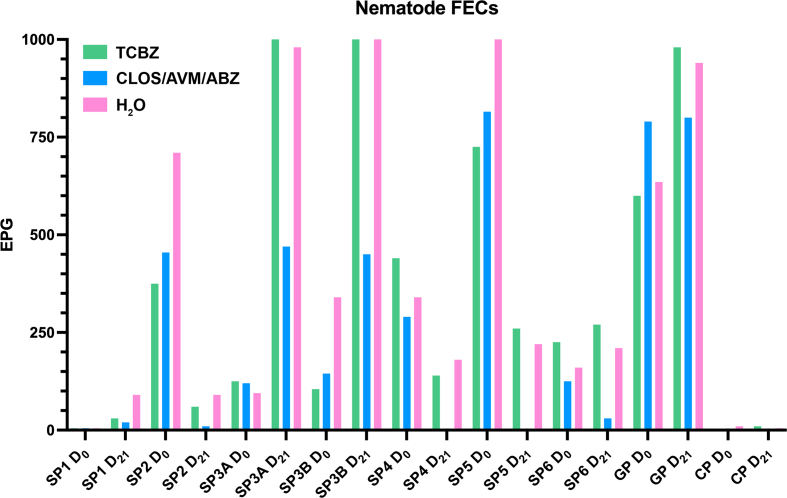


**Figure:**
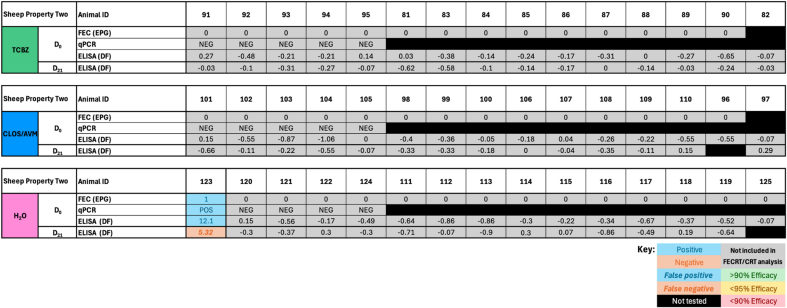


**Figure:**
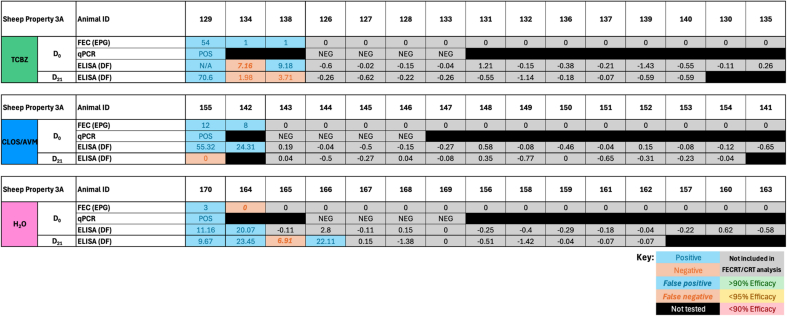


**Figure:**
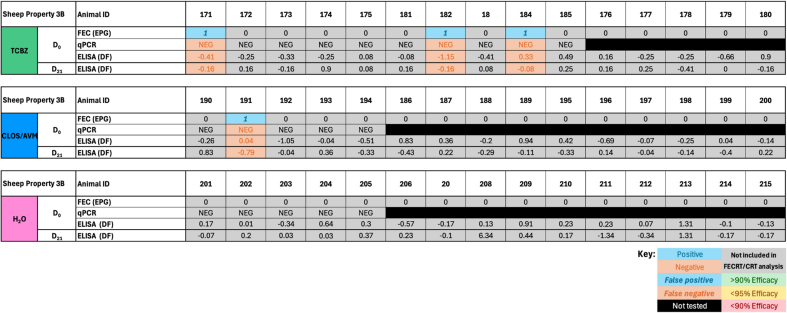


**Figure:**
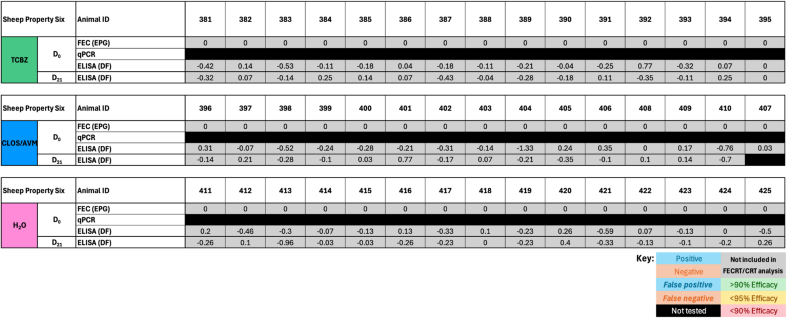


**Figure:**
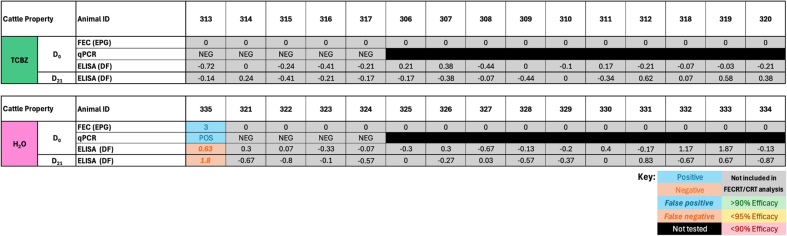


**Figure:**
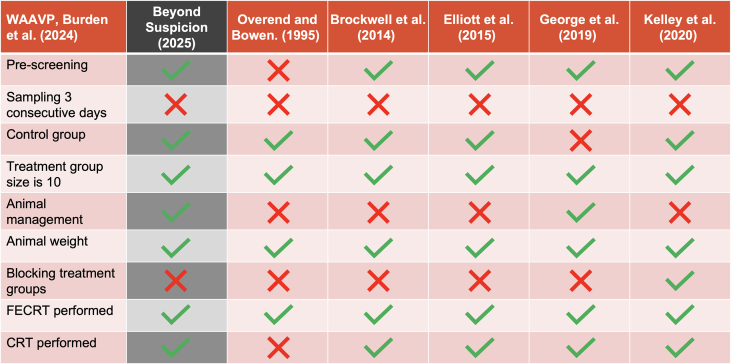


## Appendix A Supplementary data

Supplementary Figure 1. Pre-screening results for 20 farms assessed for *Fasciola hepatica* infection prior to inclusion in the field trial. Infection status was determined using pooled sedimentation faecal egg counts (FECs) and coproantigen ELISA (cELISA). The “2020–23” row indicates historical evidence of *F. hepatica* infection not diagnosed in the current study. Rows for February, April, June 1, and June 2 represent the months in 2023 when diagnostic testing was performed. “POS” indicates a positive test for *F. hepatica*; “NEG” indicates a negative result; “N/A” denotes no sample received; and “?” indicates missing information. Farms were included if infection was detected via pooled diagnostics or due to prior infection history. “YES x2” indicates that two mobs with differing exposure histories were enrolled from that farm. Farm ID numbers do not correspond to IDs used in the main text or figures

Supplementary Figure 2. Faecal egg counts (FECs) expressed as eggs per gram (EPG) on Day 0 and Day 21 for *Fasciola hepatica*-infected animals across four properties screened for triclabendazole (TCBZ) resistance in the NSW Southern Tablelands. Boxplots represent the distribution of individual FECs for each treatment group at both time points: (A) Sheep Property Four, (B) Goat Property, (C) Sheep Property One, and (D) Sheep Property Five. Treatment groups include TCBZ; green, closantel/abamectin (CLOS/AVM) or albendazole (ABZ); blue, and untreated controls (H_2_O); pink. Only animals that tested positive by FEC on Day 0 and were available for follow-up on Day 21 are included.

Supplementary Figure 3. Nematode faecal egg counts (nFECs) expressed as eggs per gram (EPG) on Day 0 and Day 21 across nine properties in the NSW Southern Tablelands. Average nFECs are shown for each treatment group in the study. Treatment groups include triclabendazole (TCBZ); green, closantel/abamectin (CLOS/AVM) or albendazole (ABZ); blue, and untreated controls (H_2_O); pink at both time points for all mobs enrolled in the study (SP = Sheep Property, GP = Goat Property, CP = Cattle Property) on Day 0. Reductions in nematode FECs over time were only expected in the CLOS/AVM or ABZ groups, as TCBZ has no activity against nematodes.

Supplementary Figure 4A-E. *Fasciola hepatica* diagnostic results for mobs that did not meet the full inclusion criteria for drug efficacy assessment. Results are presented for (A) Sheep Property Two, (B) 3A, (C) 3B, (D) Six and (E) the Cattle Property. Individual faecal egg counts (FEC) and coproantigen ELISA (cELISA) values are shown for each animal by treatment group: triclabendazole (TCBZ); green, closantel/abamectin (CLOS/AVM); blue, and untreated controls (H_2_O); pink. These mobs did not meet the W.A.A.V.P. criteria for faecal egg count reduction test (FECRT) analysis but were retained for within-mob prevalence estimation. Grey cells denote animals excluded from analysis due to negative Day 0 FECs. Negative results are shown in orange. A lack of concordance between testing methods indicating false positive (bold, italics, blue) or false negative (bold, italics, orange) results are shown. Black cells indicate animals unavailable for follow-up/insufficient sample volume on Day 21 or that were not selected for qPCR testing. Efficacy values are colour-coded: green for ≥95 %, yellow for 90–94 %, and red for <90 %. Eggs per gram (EPG), Discrimination Factor (DF)

Supplementary Figure 5. Comparison of key methodological criteria used in the current study versus Australian *Fasciola hepatica* efficacy studies published to date. Criteria are based on the most recent W.A.A.V.P. guidelines for evaluating anthelmintic efficacy in ruminants (Burden et al., 2024) and include pre-screening, control group inclusion, treatment group size, and performance of faecal egg count reduction tests (FECRT) and coproantigen reduction tests (CRT), among others. Green check marks indicate that the criterion was met; red crosses indicate those that were not.
